# The protective role of raltegravir in experimental acute lung injury *in vitro* and *in vivo*

**DOI:** 10.1590/1414-431X2022e12268

**Published:** 2022-11-04

**Authors:** Zehui Xu, Rui Ren, Wanglin Jiang

**Affiliations:** 1 School of Pharmacy, Binzhou Medical University, Yantai, China

**Keywords:** Raltegravir, Acute lung injury, NLRP3, Microvascular permeability, Claudin-18.1, VE-cadherin

## Abstract

Disruption of pulmonary endothelial permeability and associated barrier integrity increase the severity of acute respiratory distress syndrome (ARDS). This study investigated the potential ability of the human immunodeficiency virus-1 (HIV-1) integrase inhibitor raltegravir to protect against acute lung injury (ALI) and the underlying mechanisms. Accordingly, the impact of raltegravir treatment on an *in vitro* lipopolysaccharide (LPS)-stimulated human pulmonary microvascular endothelial cell (HPMEC) model of ALI and an *in vivo* LPS-induced two-hit ALI rat model was examined. In the rat model system, raltegravir treatment alleviated ALI-associated histopathological changes, reduced microvascular permeability, decreased Evans blue dye extravasation, suppressed the expression of inflammatory proteins including HMGB1, TLR4, p-NF-κB, NLRP3, and MPO, and promoted the upregulation of protective proteins including claudin 18.1, VE-cadherin, and aquaporin 5 as measured via western blotting. Immunohistochemical staining further confirmed the ability of raltegravir treatment to reverse LPS-induced pulmonary changes in NLRP3, claudin 18.1, and aquaporin 5 expression. Furthermore, *in vitro* analyses of HPMECs reaffirmed the ability of raltegravir to attenuate LPS-induced declines in VE-cadherin and claudin 18.1 expression while simultaneously inhibiting NLRP3 activation and reducing the expression of HMGB1, TLR4, and NF-kB, thus decreasing overall vascular permeability. Overall, our findings suggested that raltegravir may represent a viable approach to treating experimental ALI that functions by maintaining pulmonary microvascular integrity.

## Introduction

Acute respiratory distress syndrome (ARDS) is an extremely deadly condition for which no reliable treatments are available, leading to poor patient outcomes. Acute lung injury (ALI)-associated increases in inflammation, microvascular permeability, pulmonary edema, and neutrophil infiltration are hallmarks of ARDS (1-3). As ARDS progresses, inflammatory responses are amplified, leading to the injury and destruction of epithelial and endothelial cells at the alveolar-capillary interface and driving further inflammatory cell recruitment into the alveoli. Secondary breakdown of the epithelial and endothelial barrier layers accelerates ARDS progression and contributes to the formation of protein-rich pulmonary edema and the development of extra-pulmonary organ dysfunction (4,5). As such, approaches to reliably and effectively restoring microvascular permeability are essential to the management of ARDS. While current treatment strategies have reduced the attendant short-term mortality associated with ARDS, long-term patient morbidity and mortality remain an important concern.

Raltegravir is an oral human immunodeficiency virus-1 (HIV-1) integrase inhibitor that has been approved to treat patients infected by HIV-1 (6,7). Raltegravir alleviates endoplasmic reticulum (ER) stress and thereby suppresses HIV protease inhibitor-induced inflammation (8), while simultaneously inhibiting the nucleotide-binding oligomerization domain-like receptor 3 (NLRP3) inflammasome and thus preventing lung fibrosis (9). Whether raltegravir is capable of protecting against or treating ALI, however, remains to be established. Here, we evaluated the effects of raltegravir *in vitro* and *in vivo* using experimental models of lipopolysaccharide (LPS)-induced ALI, and we specifically examined the ability of this integrase inhibitor to prevent lung endothelial injury by suppressing NLRP3 activation and HMGB1/TLR4/NF-κB signaling in response to LPS treatment.

## Material and Methods

### Chemicals

Raltegravir (purity >98%; No. 518048-05-0) and MCC950 (purity >99%, an NLRP3 inhibitor; No. 210826-40-7) were purchased from Hanxiang Biomedical Company (China). Rabbit polyclonal antibodies specific for NLRP3, high mobility group box 1 (HMGB1), toll-like receptor 4 (TLR4), phosphorylated nuclear factor-κB (p-NF-κB), claudin 18.1, and aquaporin 5 were obtained from Abcam Biotechnology (China). Anti-vascular endothelial cadherin (VE-cadherin) was purchased from Cell Signaling Technology (China). BAY11-7082 was from Beyotime (China). Fluorescein isothiocyanate (FITC)-labeled dextran was purchased from Xiao You Biotechnology (China). A myeloperoxidase (MPO) ELISA kit was purchased from the Bio-Swamp Biological Technology Company (China).

### Cell culture

Human pulmonary microvascular endothelial cells (HPMECs, CC-2527) were purchased from the cell bank of the Chinese Academy of Sciences.

### LPS-induced ALI model establishment

Sprague-Dawley (SD) rats (∼180 g) rats were purchased (Beijing Vital River Laboratory Animal Technology Company, PR China) and allowed to acclimate to laboratory conditions for seven days, after which they were randomized into three treatment groups (n=16 per group): a control group, an LPS model group, and an LPS + raltegravir treatment group. A two-hit model of LPS-induced ALI was employed in this study as discussed previously (10). Briefly, rats in the appropriate treatment groups were intraperitoneally injected with LPS (2 mg/kg in 0.5 mL of saline). After 16 h, these same rats received an intratracheal instillation of LPS (4 mg/kg in 0.2 mL of saline). Control rats were administered equivalent volumes of saline. Animals in the LPS + raltegravir treatment group were orally administered this integrase inhibitor at a dose of 80 mg/kg once per day for 4 days. Each group of rats was then subdivided into two separate cohorts, with 8 rats per group being used to measure lung coefficient values, analyze lung histopathology, and conduct western blotting by collecting lungs on day 4 and separating them into two halves that were either fixed with 4% paraformaldehyde (PFA) or snap-frozen in liquid nitrogen. The remaining 8 rats per group were used to assess lung leakage based on an Evans blue dye extravasation assay.

### Histopathological analyses

After fixation in 4% PFA, lung samples were dehydrated, paraffin-embedded, cut into 4-μm thick sections, placed onto slides coated with poly-lysine, deparaffinized with xylene, rehydrated with an ethanol gradient, and stained using hematoxylin and eosin (H&E). The Lung Injury Scoring System used by the American Thoracic Society was used to quantify lung damage in these tissue sections (11). Scores were assigned based upon the presence of neutrophils within the alveoli, the presence of interstitial neutrophils, alveolar septal thickening, and the presence of proteinaceous debris within the airway. At least 20 randomly selected high-power fields (400× magnification) were scored in a blinded manner using a three-tiered scheme.

### Myeloperoxidase (MPO) measurements

The rat MPO ELISA kit was used to quantify MPO levels in randomly selected hydrolyzed rat lung tissue samples (100 mg) based on provided directions. First, samples were washed using PBS (0.01 M; pH 7.4). Fragments were homogenized and combined with 1 mL of prepared hydrolysate, after which samples were boiled for 20 min, spun down for 10 min at 10,000 rpm at 4°C, and supernatants were collected. Supernatant MPO levels were then measured by assessing absorbance at 450 nm and comparing absorbance values for each lung tissue sample to a constructed standard curve.

### Evans blue dye extravasation assay

The extravasation of Evans blue dye into the lung tissue was used to measure pulmonary capillary permeability. Briefly, rats received a tail vein injection of Evans blue dye (20 mg/mL). After 30 min, rats were anesthetized and perfused with 100-150 mL of 0.9% saline to remove all remaining intravascular dye from systemic circulation. Next, lung tissue samples (100 mg) were collected, and Evans blue dye was recovered by treating samples with formamide (2 mL) at 40°C for 24 h, followed by centrifugation at 400 *g* for 15 min at 4°C. Supernatants were then collected, and absorbance at 620 nm was assessed with a spectrophotometer (Poten Instrument Co., Ltd., USA).

### Immunohistochemistry

Sections of lung tissue (4 µm) were deparaffinized, rehydrated, treated with 0.01 M citric acid at 400 W in a microwave for 10 min, and then treated with 5% H_2_O_2_ in methanol for 30 min at room temperature while protected from light to quench endogenous peroxidase activity. After blocking with serum for 30 min, sections were then probed with antibodies specific for aquaporin 5 (1:500), NLRP3 (1:500), or claudin 18.1 (1:500) for 16 h at 4°C, followed by washing and incubation for 1 h with an anti-rabbit HRP-conjugated antibody at 37°C. Sections were then evaluated using a light microscope (Perkin Elmer Instrument Co., Ltd., USA).

### Western blotting

Samples of lung tissue were homogenized in radioimmunoprecipitation assay (RIPA) buffer supplemented with protease inhibitors, after which supernatant protein levels were quantified via BCA assay (12). Individual protein samples (30 μg) were then separated via 8-10% SDS-PAGE, and the resultant blots were probed with antibodies specific for aquaporin 5, VE-cadherin, claudin 18.1, NLRP3, HMGB1, TLR4, p-NF-κB, and β-actin (1:1000). Image J (NIH, USA) was then used to assess protein band density, with β-actin signal being used for normalization purposes.

### HPMEC monolayer permeability assay

HPMECs were plated onto the inner portion of a collagen-coated Transwell membrane (6.5-mm diameter polycarbonate filter with 0.4-μm pores) in a 24-well plate (12). Cells were then cultured until confluent, at which time they were treated using LPS (2 μg/mL), LPS (2 μg/mL) + raltegravir (15 μM), LPS (2 μg/mL) + MCC950 (1 μM), LPS (2 μg/mL) + MCC950 (1 μM) + raltegravir (15 μM), or were left untreated (control) for 24 h. Monolayer permeability was then assessed by adding FITC-Dextran (1 mg/mL; 40,000 Da) to the upper chamber, with PBS added to the lower chamber. Following a 20-min incubation, 10 μL-samples were collected from the lower chamber after an additional 0, 5, 30, or 90 min and after 6 h. The fluorescent signal in these samples was assessed with a Varioskan Flash Multimode Reader (Poten Instrument Co., Ltd.) at excitation and emission wavelengths of 485 and 528 nm, respectively. This signal was then used to quantify the permeability of the HPMEC monolayer, with all assays being repeated in triplicate (13).

### HPMEC culture and treatment

HPMECs were cultured in DMEM containing 10% fetal bovine serum in a humidified 37°C 5% CO_2_ incubator until approximately 80% confluent, at which time they were treated with LPS (2 μg/mL) with or without raltegravir (15 μM), the NLRP3 inhibitor MCC950 (1 μM), and/or BAY11-7028 (5 μM) for 24 h. Cells were then lysed and VE-cadherin, claudin-18.1, NLRP3, HMGB1, TLR4, and p-NF-κB protein levels were assessed via western blotting, as above.

### Immunofluorescence

HPMECs were cultured on slides and treated for 24 h with LPS (2 μg/mL) in the presence or absence of raltegravir (15 μM). Cells were then fixed with 4% PFA, blocked with 1% bovine serum albumin, and incubated overnight with anti-VE-cadherin (1:250), anti-claudin 18.1 (1:50), and anti-NLRP3 (1:500) at 4°C. Next, cells were probed for 2 h with secondary FITC-labeled goat anti-rabbit IgG (1:500) at 37°C. Mounting medium was then used to mount slides, and 4′,6-diamidino-2-phenylin-dole (DAPI) was used for nuclear counterstaining. A fluorescence microscope (model internal ID:14-PH-010, Olympus Corporation, Japan) was used for imaging. DAPI was imaged using channel 0, while claudin 18.1, NLRP3, and VE-cadherin were imaged using channel 8 (14,15).

### Statistical analysis

Wilcoxon rank-sum tests were used to compare histological scores between treatment groups. Quantitative data are reported as means±SD and were compared via two-sided ANOVAs with a significance threshold of P<0.05. When ANOVAs yielded significant results (P<0.05), Dunnett's multiple comparisons test was performed.

## Results

### Effect of raltegravir on changes in the lungs

**Figure 1:**
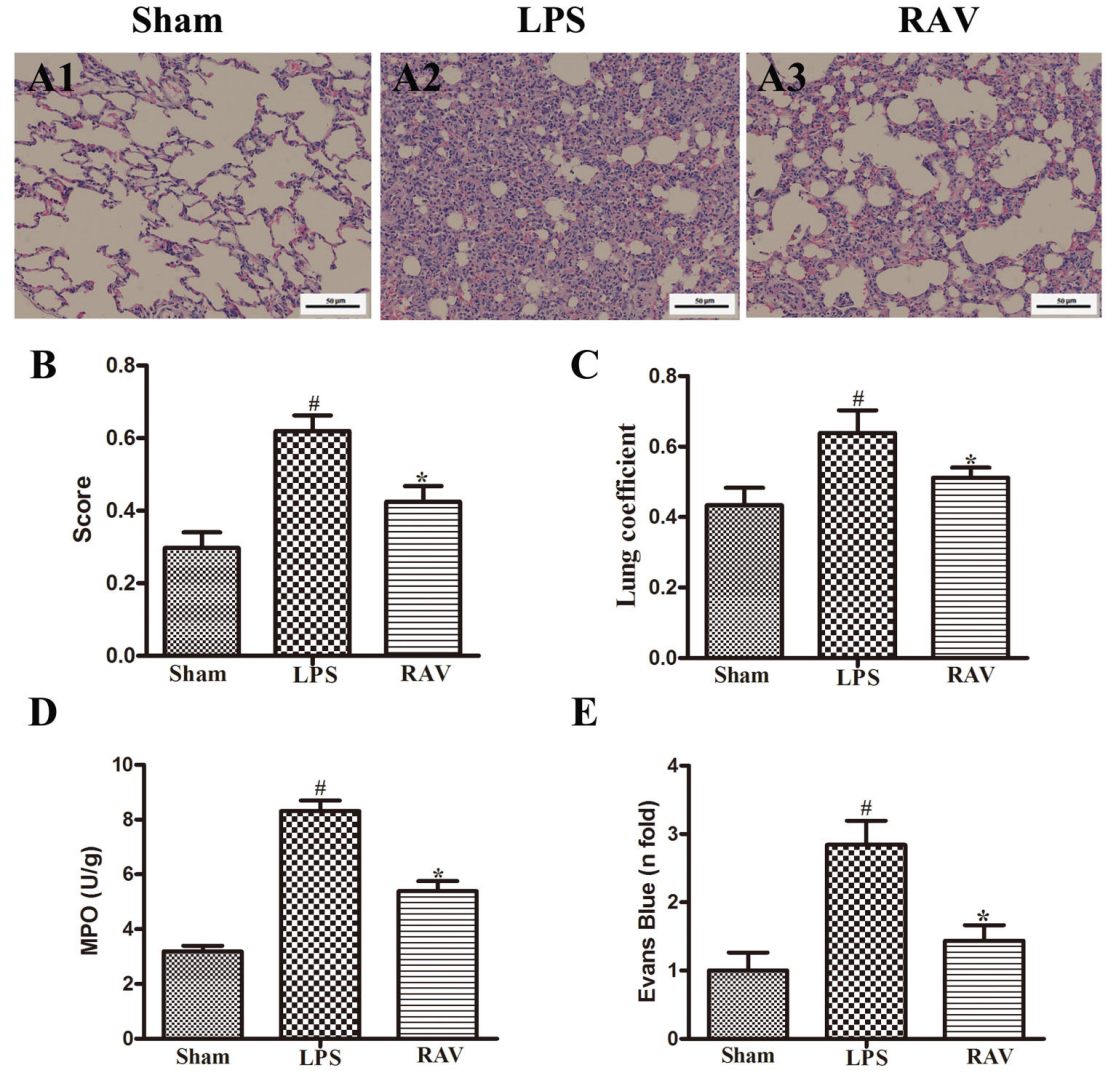
The impact of raltegravir (RAV) on acute lung injury (ALI)-associated changes in lung tissue histopathology. Representative hematoxylin and eosin (H&E) stained tissue images (**A1**-**A3**, scale bar: 50 μm). Effect of RAV treatment on histopathological scores and lung coefficients (**B** and **C**), lung MPO activity (**D**), and pulmonary capillary permeability (**E**). LPS: lipopolysaccharide. Data are reported as means±SD. ^#^P<0.01 *vs* sham; *P<0.01 *vs* the LPS group (ANOVA with Dunnett's test).

H&E staining revealed that LPS model animals exhibited significantly more alveolar destruction, inflammatory cell infiltration, diffuse alveolar and interstitial edema, thickening of the alveolar septum, and pulmonary hemorrhage than sham control animals (Figure 1A2). In contrast, the lung tissue of raltegravir-treated model animals was more similar to that of control animals, with significant reductions in edema, neutrophil invasion, and disruption of pulmonary morphology (Figure 1A3). These data clearly indicated that raltegravir can significantly attenuate LPS-induced lung injury in rats.

Lung coefficient values, defined as the ratio of lung weight to body weight, were additionally measured in these animals as a means of quantitatively gauging the severity of pulmonary edema (16), with more severe lung injury and associated edema being correlated with higher lung coefficient values. Consistent with such a model, we found that raltegravir-treated rats exhibited significantly decreased lung coefficient values compared with LPS model animals (Figure 1C).

MPO is a key mediator of neutrophil antimicrobial activity involved in the phagocytic uptake of pathogens, but it can also be measured to assess lung injury severity. We found that MPO levels in raltegravir-treated rats were significantly lower than those in LPS model animals (Figure 1D).

### Effect of raltegravir on pulmonary vascular permeability

We next utilized an Evans blue dye extravasation assay to gauge pulmonary vascular permeability in our ALI model rats by assessing the ability of this dye to permeate into lung lavage fluid following its intravenous administration (17). LPS-induced increases in vascular leakage were found to be significantly suppressed following raltegravir treatment (Figure 1E).

### Effect of raltegravir on *in vivo* protein expression

**Figure 2:**
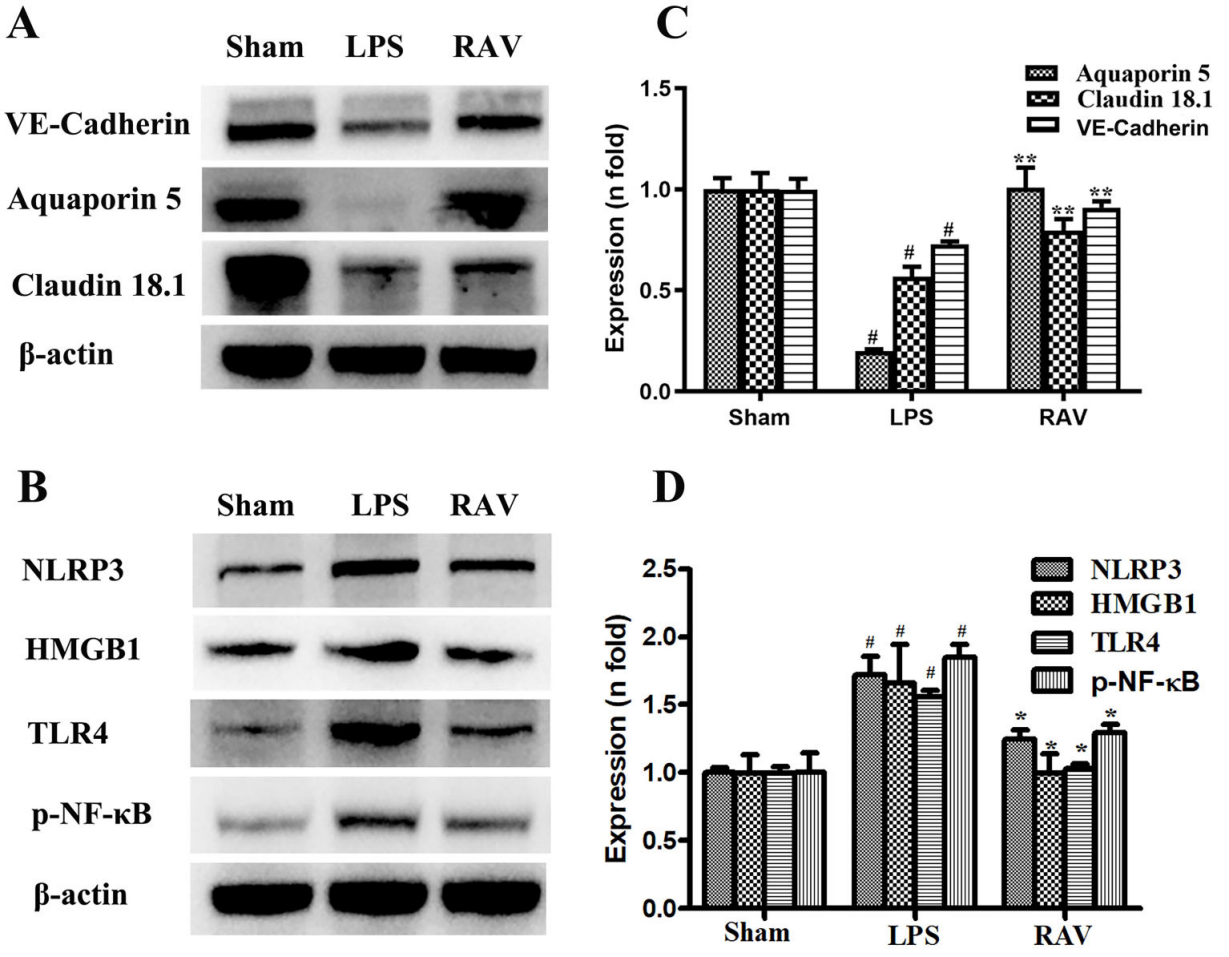
The impact of raltegravir on *in vivo* protein expression (**A**-**D**). The expression of aquaporin 5, VE-cadherin, claudin 18.1, NLRP3, HMGB1, TLR4, and p-NF-kB was assessed. LPS: lipopolysaccharide; RAV: raltegravir. Data are reported as means±SD. ^#^P<0.01 *vs* sham; *P<0.01, **P<0.001 *vs* the LPS group (ANOVA with Dunnett's test).

Claudin 18.1 is an alveolar tight junction protein expressed in endothelial and alveolar epithelial cells that is related to endothelial permeability and alveolar-capillary barrier integrity (18,19). Aquaporin 5 is similarly a marker associated with epithelial barrier integrity (20), whereas NLRP3 is a key innate immune sensor protein associated with inflammatory processes in the vascular endothelium (21). Relative to sham control rats, LPS model animals exhibited significant reductions in claudin-18.1, aquaporin 5, and VE-cadherin protein levels, which were increased in raltegravir-treated animals (Figure 2A and C). In contrast, NLRP3, HMGB1, TLR4, and p-NF-κB protein levels were significantly higher in LPS model animals, while levels of these proteins were lower in raltegravir-treated rats (Figure 2B and D).

**Figure 3:**
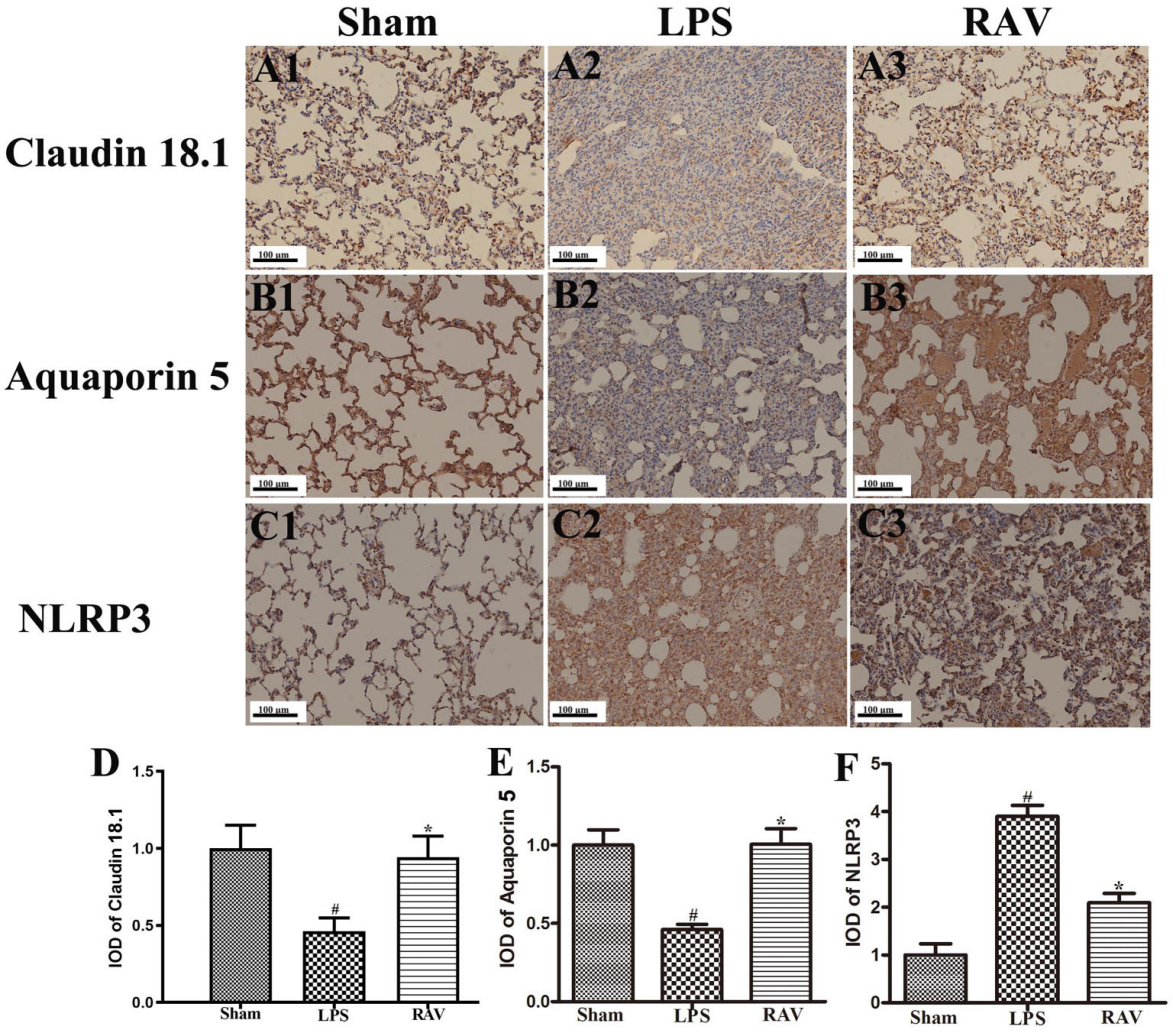
Impact of raltegravir on LPS-associated changes in pulmonary claudin 18.1, aquaporin 5, and NLRP3 expression (**A-F**). Immunohistochemical staining was used to assess changes in claudin 18.1, aquaporin 5, and NLRP3 expression following lipopolysaccharide (LPS) and raltegravir (RAV) treatment (scale bar: 100 μm). Data are reported as means±SD. ^#^P<0.01 *vs* sham; *P<0.01 *vs* the LPS group (ANOVA with Dunnett's test).

Claudin 18.1 and NLRP3 are closely associated with the onset and severity of ALI. In immunohistochemical analyses, we found that cytoplasmic claudin 18.1 staining was clearly evident in lung tissue samples from sham control rats (Figure 3A1 and D), whereas these levels were reduced in samples from LPS model animals (Figure 3A2 and D), and were significantly higher in raltegravir-treated rat lung samples (Figure 3A3 and D). Aquaporin 5 immunohistochemical staining results yielded the same patterns as those observed for claudin 18.1 (Figure 3B1-B3 and E). Lung tissue samples from LPS-treated model rats exhibited strong NLRP3 staining (Figure 3C1, 3C2, and F), whereas raltegravir treatment reduced the frequency and intensity of NLRP3 staining in LPS-treated animals, although not to levels matching those observed in sham control animals (Figure 3C2, 3C3 and F).

Overall, our *in vivo* results suggested that raltegravir treatment enhanced pulmonary aquaporin 5, claudin 18.1, and VE-cadherin expression, consistent with increased microvascular integrity, while simultaneously suppressing NLRP3, HMGB1, TLR4, and p-NF-κB expression. This indicates that raltegravir can bolster pulmonary microvascular integrity and suppress inflammation in a rat model of ALI.

### Effect of raltegravir on endothelial dysfunction *in vitro*

**Figure 4:**
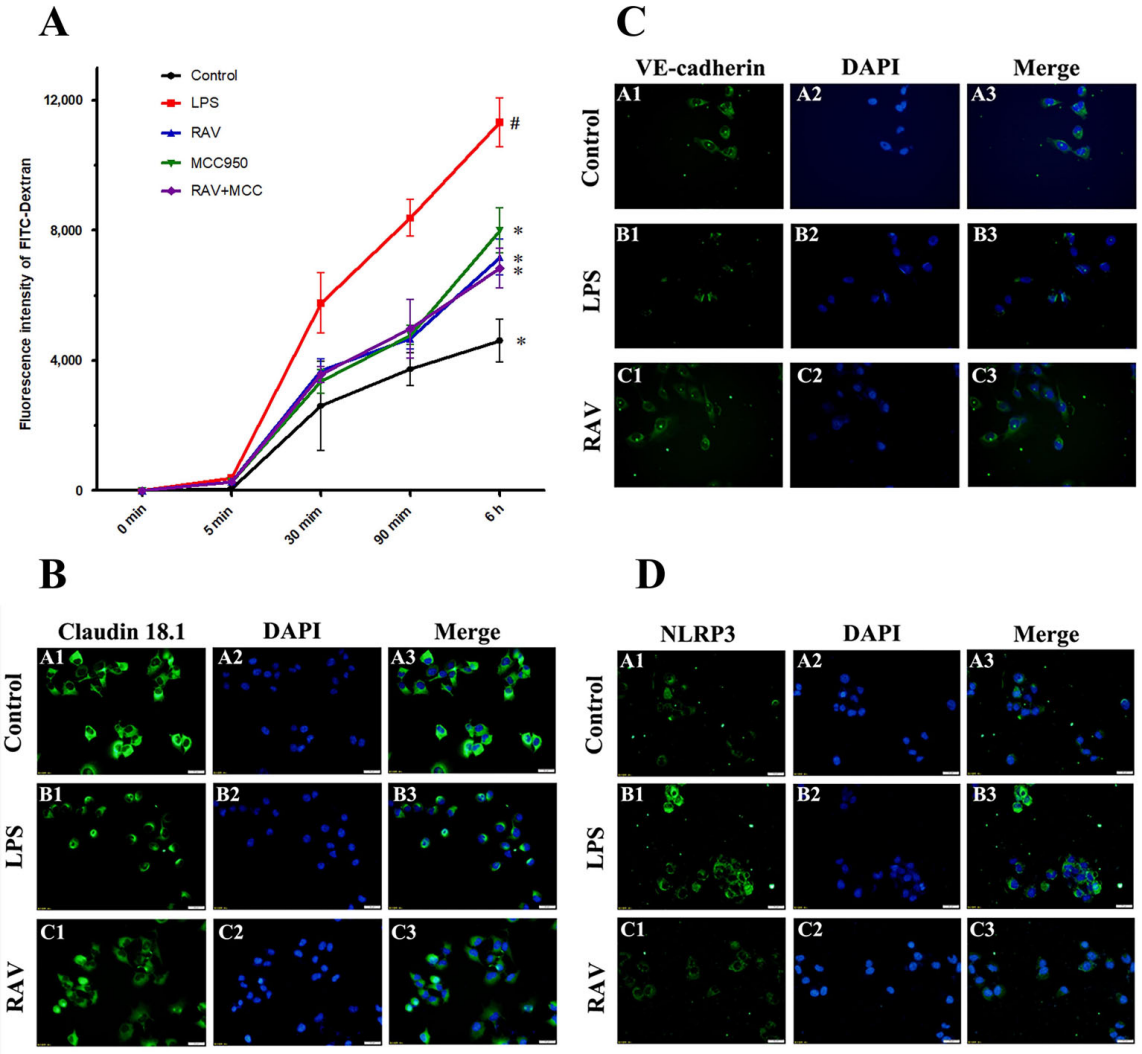
The impact of raltegravir (RAV) on endothelial cell permeability and claudin 18.1, VE-cadherin, and NLRP3 expression. Magnification: ×40, scale bar: 20 μm. FITC-dextran passage across the HPMEC monolayer was assessed over a 6-h period, with data being assessed based on cumulative FITC-dextran levels over time (**A**). Confluent human pulmonary microvascular endothelial cell (HPMEC) monolayers were stained for claudin 18.1 (**B**), VE-cadherin (**C**), and NLRP3 (**D**). Data are reported as means±SD. ^#^P<0.01 *vs* the control group; *P<0.01 *vs* the LPS group (ANOVA with Dunnett's test).

To confirm and expand upon these *in vivo* findings, we next utilized an *in vitro* HPMEC model system. Low-molecular-weight FITC-dextran was used to assess LPS-treated HPMEC monolayer permeability in the presence or absence of raltegravir. LPS enhanced endothelial monolayer permeability and consequent FITC-dextran leakage, whereas raltegravir treatment enhanced monolayer integrity and reduced the permeability of these LPS-treated HPMECs (Figure 4A).

To confirm that the ability of raltegravir to alleviate LPS-induced endothelial dysfunction was linked to the strengthening of cell-cell junctions, we next measured VE-cadherin and claudin 18.1 expression levels in these HPMECs via western blotting and immunofluorescent (IF) staining. IF staining revealed that LPS treatment markedly suppressed VE-cadherin and claudin 18.1 expression in treated HPMECs, whereas such suppression was largely reversed in raltegravir-treated HPMECs (Figure 4B and C). In addition, LPS treatment markedly induced NLRP3 activation within these cells, whereas raltegravir pretreatment disrupted such LPS-induced NLRP3 expression (Figure 4D).

**Figure 5:**
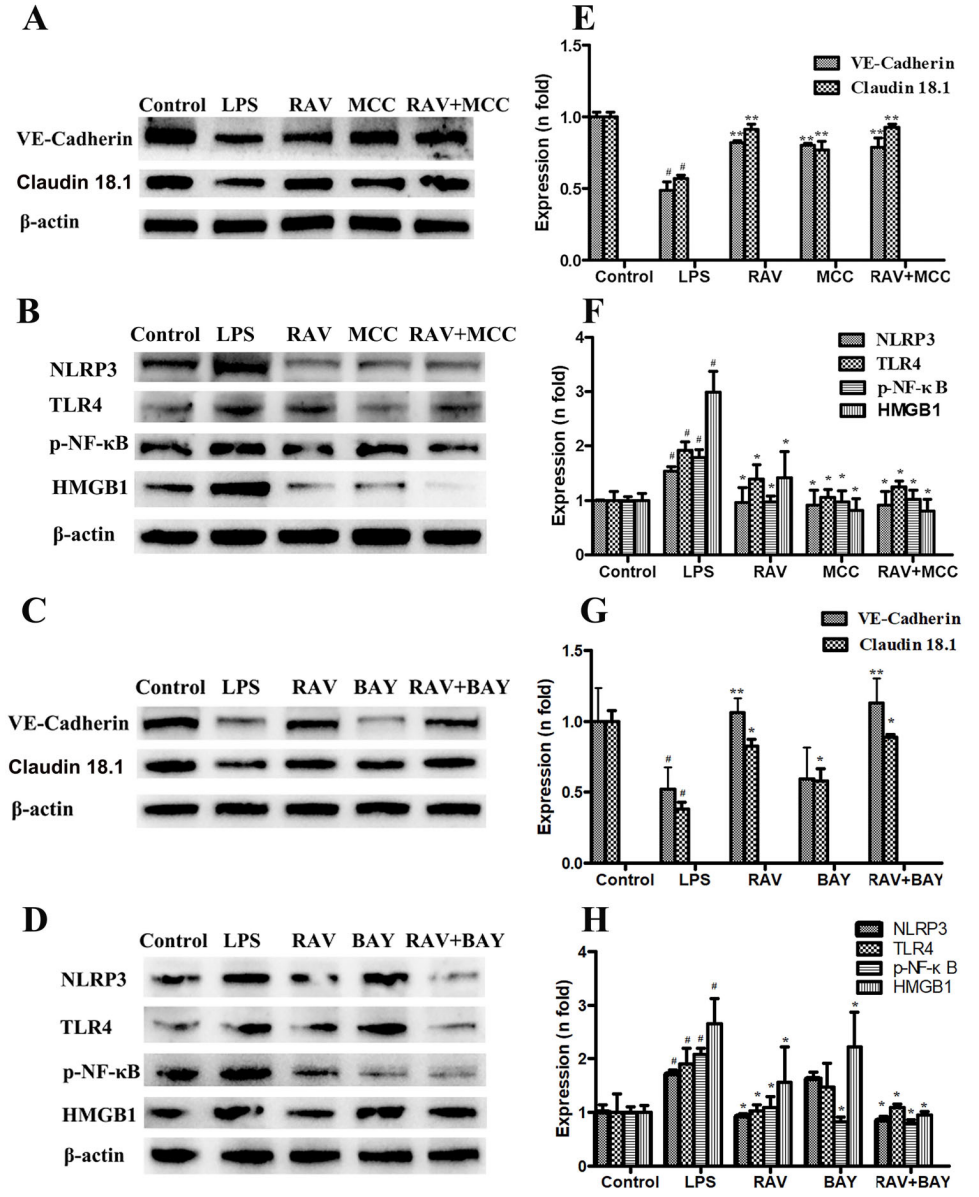
Impact of raltegravir (RAV) on protein expression in human pulmonary microvascular endothelial cell (HPMECs) treated with lipopolysaccharide (LPS), MCC950, and/or BAY11-7082. The expression of VE-cadherin, claudin 18.1, NLRP3, HMGB1, TLR4, and p-NF-κB was assessed. Data are reported as means±SD. ^#^P<0.01 *vs* the control group; *P<0.01, **P<0.001 *vs* the LPS group (ANOVA with Dunnett's test).

To better understand how raltegravir alleviates LPS-induced endothelial dysfunction and whether such activity is linked to NLRP3 inflammasome or NF-κB activity, these cells were treated with the NLRP3 inhibitor MCC950 or the NF-κB inhibitor BAY11-7082. Western blotting confirmed that LPS treatment enhanced NLRP3 activation (Figure 5B and F) and significantly attenuated VE-cadherin and claudin 18.1 expression in HPMECs (Figure 5A and E). Raltegravir pretreatment prevented these LPS-induced changes in NLRP3, VE-cadherin, and claudin 18.1 expression (Figure 5A, B, E, and F). MCC950 treatment decreased NLRP3 expression and increased VE-cadherin and claudin 18.1 expression (Figure 5A, B, E, and F), whereas trends were identical in the MCC950 + raltegravir group, with no significant differences between this treatment group and the MCC950 group or the raltegravir group (Figure 5A, B, E, and F). Furthermore, p-NF-κB expression was decreased in LPS-stimulated HPMECs that had been treated with BAY11-7082, whereas NLRP3 expression was not reduced in these cells, nor were VE-cadherin and claudin 18.1 expression increased. BAY11-7082 + raltegravir treatment did not further reduce p-NF-κB expression relative to BAY11-7082 alone (Figure 5C, D, G, and H). Together, these results indicated that raltegravir reduced endothelial permeability and alleviated endothelial dysfunction via inhibiting NLRP3 activation.

## Discussion

ALI is a common yet potentially serious clinical condition that can in some cases progress to ARDS. These dangerous conditions are characterized by the destruction of normal lung tissue structure as a consequence of severe pulmonary inflammation (22). In clinical settings, ALI/ARDS patients exhibit hypoxemia and decreased lung compliance that are associated with prolonged hospitalization and high mortality rates (23). The exact pathological basis for ALI is largely unclear, with pneumonia, extrapulmonary sepsis, and aspiration being the clinical risk factors most often associated with this condition. ARDS rates are also linked to specific demographic and environmental risk factors. The most effective treatments for ALI include specific drugs and mechanical ventilation, and while there have been recent advances in the development of stem cell- and targeted therapy-based treatments for this condition, mortality rates nonetheless remain high (24). It is thus vital that novel treatments for ALI be developed. Diffuse alveolar damage (DAD) and consequent pulmonary edema is a hallmark of ALI/ARDS, and as such, we conducted the present study to assess whether alleviating such high permeability and pulmonary edema was a viable approach to ameliorating ALI-induced lung damage.

The analyses revealed that raltegravir treatment alleviated LPS-induced changes in lung index values, MPO levels, pathological damage, and pulmonary vascular permeability while simultaneously enhancing the expression of endothelial barrier-related junction proteins. These data thus enabled us to conclude that raltegravir can protect against LPS-induced lung injury *in vivo.*

To evaluate the mechanistic basis whereby raltegravir protected against LPS-induced ALI, we studied HPMECs *in vitro* through western blotting, immunofluorescent staining, and monolayer permeability assays. Endothelial cells form a semi-permeable barrier that controls the ability of cells, nutrients, and other compounds to enter into surrounding tissues (25). Endothelial permeability and associated barrier integrity are thus critical regulators of tissue homeostasis such that the disruption of this functionality can cause vascular hyper-permeability, promoting chronic inflammation, and diseases including asthma, edema, sepsis, and ARDS (26). Endothelial permeability is controlled by a range of intra- and intercellular pathways, with intercellular pathways being dependent upon the regulation of endothelial cell-cell junction opening and closing. Endothelial cells form both adherens junctions (AJs) and tight junctions (TJs) (27,28), which are composed of VE-cadherin and junctional adhesion molecules such as claudin 18.1, respectively. LPS promoted pulmonary vascular hyperpermeability at least in part by driving claudin 18.1 and VE-cadherin internalization and degradation. Importantly, raltegravir reversed such LPS-induced AJ and TJ disruption, thereby maintaining endothelial barrier integrity (29). Monolayer permeability assay results also indicated that raltegravir treatment prevented LPS-driven increases in HPMEC monolayer permeability. Raltegravir can thus alleviate endothelial dysfunction by suppressing vascular permeability.

The NLRP3 inflammasome is a key mediator of inflammatory responses that are closely tied to a range of pathological processes within the vascular system (30). We have previously identified NLRP3 as a potential raltegravir target in the context of lung fibrosis (5). As such, in this study we hypothesized that raltegravir-mediated NLRP3 inhibition may be a major mechanism whereby this integrase inhibitor can protect vascular endothelial cells against ALI-associated damage. To test this, we assessed NLRP3 inflammasome activation and changes in vascular permeability in the presence or absence of an NLRP3 inhibitor (MCC950) or an NF-κB inhibitor (BAY11-7082). The results revealed that raltegravir prevented LPS-induced vascular hyper-permeability, at least in part, by inhibiting NLRP3 activation, thus ameliorating ALI-associated pathological damage.

Inflammation is a critical driver of ALI development (31). HMGB1 is a nuclear transcription factor that can signal through TLR4 when exposed to the extracellular space by activating TLR4 and stimulating NF-κB activation (32,33). This HMGB1/TLR4/NF-κB signaling pathway is thus a key mediator of ALI/ARDS (1). Herein, we found that raltegravir treatment reduced HMGB1 and p-NF-κB levels in the lungs of LPS-treated rats, and we confirmed the ability of this integrase inhibitor to suppress HMGB1 secretion and p-NF-κB expression in LPS-stimulated HPMECs *in vitro*. These findings indicated that raltegravir enhanced endothelial barrier integrity, thereby reducing ALI-associated inflammation via modulating HMGB1/TLR4/NF-κB signaling and inhibiting NLRP3 activation. However, these findings are limited by the fact that no studies of oxygenation, saturation, or pulmonary mechanics were performed.

### Conclusions

In conclusion, our data indicated that raltegravir reversed the breakdown of VE-cadherin- and claudin 18.1-containing endothelial adherens junctions both *in vitro* and *in vivo,* thereby restoring endothelial barrier integrity and reducing ALI-associated vascular permeability. At a molecular level, raltegravir enhanced such microvascular integrity via suppressing HMGB1/TLR4/NF-κB signaling and NLRP3 activation. As such, raltegravir may represent a promising therapeutic tool for the treatment and management of ALI/ARDS.
